# Comment on Rauchman et al. Mild-to-Moderate Traumatic Brain Injury: A Review with Focus on the Visual System. *Neurol. Int.* 2022, *14*, 453–470

**DOI:** 10.3390/neurolint14040068

**Published:** 2022-10-21

**Authors:** Nir Erdinest, Naomi London

**Affiliations:** 1 Department of Ophthalmology, Hadassah-Hebrew University Medical Center, Jerusalem 9112001, Israel; 2 Private Practice, Jerusalem 9422805, Israel

This letter is regarding the article, “Mild-to-Moderate Traumatic Brain Injury: A Review with Focus on the Visual System” [1], which we read with great interest. The article was published in the *Neurology International* journal, Volume 14, on 30 May 2022. The article authors are Steven H. Rauchman, Jacqueline Albert, Aaron Pinkhasov and Allison B. Reiss.

The current review discussed the pathophysiology and difficulty of moderate traumatic brain injury (TBI) and the effects of TBI on the visual system and visual perception. In addition, the authors supply several tools for ophthalmologic assessment. As optometrists, we felt compelled to add some information directly related to the visual symptoms, evaluation and treatment briefly mentioned in this review that can directly impact the positive collaboration between care providers, and ultimately, benefit the patients greatly.

The authors mentioned the importance of evaluating oculomotor movements, vergences and accommodation. Likewise, the sentence, “The treatment of the visual symptoms and findings of TBI involve the prescription of eyeglasses with tints and prism combinations, bi-nasal occlusion as well as light-filtering lenses. Interdisciplinary care is needed to get the best outcomes of rehabilitation for these patients”, encompasses the treatment discussed in this manuscript. As suggested by the authors, the most prevalent additional diagnoses were accommodative insufficiency and eye movement issues. The advantage of collaborative rehabilitation, specifically with optometrists trained in neuro-optometric sciences, can be expanded on due to the interaction between the senses, yet we will limit this communication to highlight one diagnosis not mentioned.

This article was a review and clearly should not expand too extensively, yet studies have shown that convergence insufficiency (CI) is prevalent in at least 47% of TBI [2] or concussion [3] (a mild form of TBI, which accounts for 80% of the U.S. recognized cases patients [1]), but this was not mentioned save for a brief comment to check vergences.

CI is such a widespread diagnosis that so profoundly impacts near function, that effective treatment has been thoroughly researched. Although the abovementioned utilized therapies are effective for various symptoms, we propose including office-based vision therapy as an important tool to include in the treatment armament. Office-based therapy specifically for CI is implemented after a thorough assessment of the visual binocular system, including, but not exclusively, the vergence, accommodative and oculomotor systems, such as amplitude, facility, sustainability and recovery after disruption, of the various skills. In-office therapy includes preplanned regimens implemented at set interval visits to the office with a trained designated professional (usually weekly). Though the goals and stages for CI therapy are somewhat systematic, therapy is constantly modified and customized to each individual. The patient internalizes, implements and reinforces the skills learned at home between office sessions. Office-based vision therapy programs for TBI patients are very comprehensive and not just focused on treating the CI independently. The vision therapy regimen is continuously modified for each individual’s symptoms, progress and manifestations, and, as the authors aptly describe, ultimate success is achieved with interprofessional collaboration. The positive effect of vision therapy has been exhibited in both young patients and adults after TBI. Therefore, we feel it would be beneficial for the esteemed readership of this fine review to be aware of this additional positive impact they can have on their patients [3,4,5].
